# Characterization of Ceramide Kinase from Basolateral Membranes of Kidney Proximal Tubules: Kinetics, Physicochemical Requirements, and Physiological Relevance

**DOI:** 10.3390/ijms262110373

**Published:** 2025-10-24

**Authors:** Gloria M. R. S. Grelle, Lindsey M. P. Cabral, Fernando G. Almeida, Giovane G. Tortelote, Rafael Garrett, Adalberto Vieyra, Rafael H. F. Valverde, Celso Caruso-Neves, Marcelo Einicker-Lamas

**Affiliations:** 1 Laboratório de Biomembranas, Instituto de Biofísica Carlos Chagas Filho, Universidade Federal do Rio de Janeiro, Ilha do Fundão, Rio de Janeiro 21941-902, Brazil; gloriagrelle@biof.ufrj.br (G.M.R.S.G.); lindseycabral@biof.ufrj.br (L.M.P.C.); fgalmeida@biof.ufrj.br (F.G.A.); valverde@biof.ufrj.br (R.H.F.V.); 2 Laboratório de Metabolômica, LADETEC, Instituto de Química, Universidade Federal do Rio de Janeiro, Ilha do Fundão, Rio de Janeiro 21941-598, Brazil; rgarrett@iq.ufrj.br; 3 Department of Pediatrics, School of Medicine, Tulane University, New Orleans, LA 70112, USA; gtortelote@tulane.edu; 4 Laboratório de Físico-Química Biológica Aída Hasson Voloch, Instituto de Biofísica Carlos Chagas Filho, Universidade Federal do Rio de Janeiro, Ilha do Fundão, Rio de Janeiro 21941-902, Brazil; avieyra@biof.ufrj.br; 5 Centro Nacional de Biologia Estrutural e Bioimagem—CENABIO, Universidade Federal do Rio de Janeiro, Ilha do Fundão, Rio de Janeiro 21941-902, Brazil; 6 Laboratório de Sinalização Celular, Instituto de Biofísica Carlos Chagas Filho, Universidade Federal do Rio de Janeiro, Ilha do Fundão, Rio de Janeiro 21941-902, Brazil; caruso@biof.ufrj.br

**Keywords:** ceramide kinase, ceramide-1 phosphate, kidney, proximal tubules, basolateral membranes, bioactive sphingolipids

## Abstract

Ceramide kinase (CerK) catalyzes the phosphorylation of ceramide to ceramide-1-phosphate (C1P), a bioactive sphingolipid with diverse signaling roles. While CerK has been identified in several cellular compartments, its presence and functional significance in kidney proximal tubules remain unexplored. Herein, we report the first characterization of CerK activity in basolateral membranes (BLMs) from porcine proximal tubule cells. We demonstrate that BLM fractions contain neutral and acidic sphingomyelinases, providing local substrate for CerK, which efficiently generates C1P under physiological pH (6.5–7.2) and temperature (30–37 °C) conditions. Enzyme activity was stimulated by cAMP in a protein kinase A-dependent manner but was not affected by angiotensin II. Lipidomic analysis confirmed the presence of C1P in human proximal tubule (HK-2) cells under basal conditions and revealed changes during ischemic stress. Transcriptomic analysis of kidney biopsies from patients with chronic kidney disease (CKD) further uncovered coordinated remodeling of sphingolipid metabolism genes, with increased expression of ceramidases (ASAH1 and NAAA) and downregulation of ceramide synthases (CERS4, CERS5), consistent with adaptive regulation of the Cer/CerK/C1P axis. Together, these findings identify for the very first time CerK activity in renal BLM, establish its biochemical requirements, and highlight its potential role in modulating transporter function and sphingolipid signaling in physiology and kidney disease.

## 1. Introduction

The basolateral membrane (BLM) of kidney proximal tubule epithelial cells functions as an important signal-transducing structure, constituting an interesting model for the study of ion and solute transport and molecules associated with related signaling pathways, such as receptors, protein kinases, and bioactive lipids [1,2,3,4,5]. The basolateral membrane anchors primary active transporters, such as the Na^+^+K^+^-ATPase, the second Na^+^ pump or Na^+^-ATPase, and the plasma membrane Ca^2+^-ATPase, which colocalizes within the BLM with a wide variety of receptors for different hormones and autacoids [1,6,7]. The activation of these receptors leads to the production of secondary messengers capable of triggering specific functional modifications in cellular metabolism, regulating the ionic flow through the two membranes (apical and basolateral) and the epithelium as a whole [8,9,10].

The determinant role played by some lipids in the modulation of membrane dynamics has been thoroughly documented. For example, it has been demonstrated that very low proportions of messenger lipids such as ceramides, diacylglycerol, some types of fatty acids, sphingolipids, glycolipids, and their metabolic products cause changes in the molecular organization of mixed interfaces with phospholipids. This can extend to supramolecular levels and induce topological changes that affect the formation of segregated domains of different phase states, thickness, and interfacial curvature that can participate in signal transduction mediated by phospholipase A2 (PLA2), phospholipase C (PLC), and sphingomyelinases [11,12,13,14].

Sphingolipid metabolism involves numerous metabolites localized in various cellular compartments and performing distinct intracellular functions. This diverse class of lipids is composed of a long-chain sphingoid base—sphingosine (Sph)—that is linked to a fatty acid by an amide bond (hydrophobic region) and presents a hydrophilic region consisting, in the simplest case, of a hydroxyl group, as observed in ceramide. The fatty acid composition of sphingolipids is variable, but palmitic acid (C16:0) and stearic acid (C18:0) are often present. Sphingosine-1 phosphate (S1P) and ceramide (Cer) are the most studied sphingolipid metabolites, and their biological functions in cells have been extensively demonstrated [15,16,17]. Their seemingly opposite functions within cells led to the concept that they are part of a “rheostat” of cell fate, with S1P-promoting mechanisms leading to cell survival, inflammation, angiogenesis, and cell invasion, and ceramide-promoting mechanisms leading to apoptosis, cell cycle arrest, and senescence [18,19,20]. Cell fate is ultimately determined not only by the intracellular levels of S1P, Cer, and other sphingolipid metabolites but also by their compartmental localization, turnover, and remodeling, here including sphingolipid metabolizing enzymes such as sphingomyelinases (SMases), ceramidases (Cdases), sphingosine kinases (SPK), lyases, and ceramide kinases (CerK), a complex and intriguing scenario that enable the reconsideration of just a simple balance between S1P and Cer levels in the “rheostat” concept [20,21].

Ceramide is the centerpiece of sphingolipid metabolism and is produced either via de novo synthesis or via turnover of other sphingolipids [16,17]. De novo Cer synthesis starts at the endoplasmic reticulum with sequential and coordinated steps involving different intermediates that allow the formation of dihydroceramide, which is then converted to Cer by the different ceramide synthases. Alternatively, Cer can be generated directly at the plasma membrane as well as at other subcellular locations through the hydrolysis of sphingomyelin (SM) to Cer and phosphocholine through the action of SMases [16,22,23]. Cer can either be converted to Sph by CDase or to ceramide-1-phosphate (C1P) by CerK. Nonetheless, it can be turned back to SM by sphingomyelin synthase [15,23,24]. CerK, the enzyme that catalyzes the conversion of Cer to C1P, was cloned in the early 2000s [25] and is normally associated with the trans-Golgi network, although different pieces of evidence suggest that it can be widespread within tissues and cell types [19,26,27,28].

Cer is involved in cell signaling events, with several cellular effects, such as: proliferation, cell survival, differentiation, apoptosis, etc. [16,29], involving modulation of phosphatases (PP1A and PP2A), kinases (PKA and PKC), phospholipase (PLD), and Ras [3,5,24,30,31,32], cellular actions that depend on the size of the Cer hydrocarbon chain and the cell type studied. C1P, the phosphorylated derivative of Cer, is also related to different and important cellular effects; for example, it blocks apoptosis, increases phagocytosis, mediates inflammatory responses, and is mitogenic, in addition to modulating kinases (PKA, PKC, ERK, and PI3K), lipases (PLD and cPLA2 alpha), and inhibiting acid SMases [3,4,5,17,24,30,33]. In addition to its action as a signaling molecule, Cer plays a decisive role in the organization of regulatory microdomains present in plasma membranes, called rafts or signaling platforms, which are enriched in sphingolipids and cholesterol [34,35]. These lipid microdomains concentrate elements involved in cell signaling cascades, increasing the efficiency of the cellular response [34]. Thus, the generation of Cer molecules leads to significant changes in the organization of the plasma membrane with increased formation of small Cer-rich microdomains, which are capable of spontaneously binding to other microdomains, resulting in the so-called signaling platforms [36,37,38]. In this way, Cer-rich microdomains play a crucial role in facilitating and enhancing signaling processes through various cellular receptors. This amplification leads to the activation of a cellular machinery, ultimately resulting in specific cellular responses [35,39].

Higher expression of CerK is observed in the heart, brain, skeletal muscle, liver, and kidneys. A high similarity was observed between the catalytic region of CerK and that of SPK [25], a lipid kinase that plays a role in balancing two bioactive sphingolipids: Sph and S1P.

Since we have previously demonstrated C1P formation within the BLM [3], and also the modulatory potential of this bioactive sphingolipid in the modulation of at least two important ion transporters that play key roles in renal physiology and are placed in the BLM from kidney proximal tubules [3,4,5], we aimed to characterize BLM CerK activity, as it is closely involved in modulating the levels of Cer and C1P, with the balance between these two sphingolipids being extremely relevant to different cellular responses [16,18,20]. Additionally, once the Cer/CerK/C1P axis is strategically established in the BLM, we aim to determine the physiological relevance of this pathway involving bioactive sphingolipids in renal physiology and pathophysiology.

## 2. Results

The modulatory action of the bioactive sphingolipids Cer and C1P on the BLM Ca^2+^ and Na^+^ transporting ATPases, through triggering different BLM-associated protein kinases as presented by our group, led us to investigate the presence of Cer and C1P generation machinery (SMases and CerK, respectively) within the BLM.

**Figure 1:**
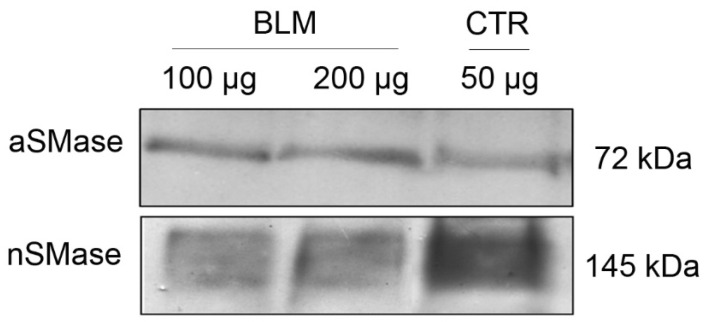
Presence of acidic and neutral sphingomyelinases in the basolateral membrane from kidney proximal tubules. The immunodetection of acidic and neutral SMases was assayed as described in Section 4, in the presence of 100 µg and 200 µg of protein. Pig kidney homogenate (50 µg) was used as a positive control (CTR) for acidic sphingomyelinase, and pig liver homogenate (50 µg) for neutral sphingomyelinase.

Figure 1 shows the presence of SMases isoforms in basolateral membrane-enriched (BLM) fractions from porcine renal cortex. Immunoblotting with an anti-acid SMase (aSMase; SMPD1) antibody revealed a faint band at approximately 70 kDa, consistent with the lysosomal enzyme (Figure 1, upper panel). This low signal indicates only minimal contamination with lysosomal components, supporting the enrichment of plasma membrane structures in the BLM preparation. In contrast, the anti-neutral SMase (nSMase2; SMPD3) antibody detected a robust band at approximately 145 kDa, which likely represents the dimeric form of SMPD3 (Figure 1 lower panel).

Once the presence of acidic and neutral SMases in the BLM was demonstrated, our next step was to investigate the presence of a BLM-associated CerK, which would be possible by detecting the phosphorylation of Cer converting it to C1P, thus evidencing the presence of a complete machinery capable of rapid local generation of both Cer and C1P. It is worth mentioning that we found no data in the literature demonstrating the presence of a CerK in the BLM.

**Figure 2:**
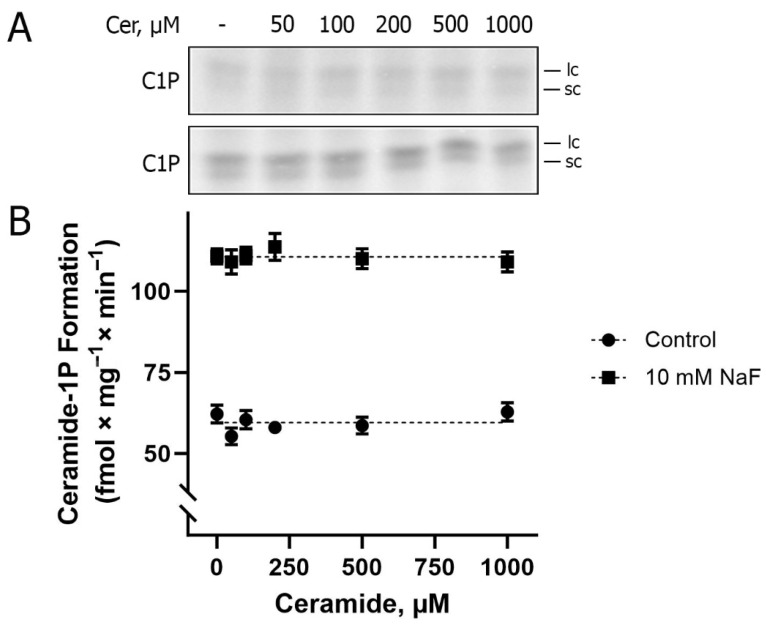
Effect of exogenous ceramide and phosphatase inhibition on Ceramide-1P formation in basolateral membrane-enriched fractions from porcine kidney cortex. (**A**) Representative TLC phosphor screen autoradiograms showing ceramide-1-phosphate (C1P) formation in response to increasing concentrations of exogenous ceramide (0–1000 µM) in the absence (top panel) or presence (bottom panel) of 10 mM NaF (phosphatase inhibitor). The labels “lc” and “sc” on the autoradiograms refer to longer-chain and shorter-chain ceramide-1-phosphate species, respectively. (**B**) Quantification of CerK activity expressed as C1P formation (fmol Pi × mg^−1^ × min^−1^). NaF treatment caused a robust increase in C1P formation relative to control (*p* < 0.0001), whereas increasing exogenous ceramide concentrations had no significant effect on C1P formation in either condition. Statistical analysis by two-way ANOVA revealed a highly significant main effect of NaF (F(1, 48) = 860.45, *p* < 0.0001), but no significant effect of ceramide concentration (*p* = 0.963) and no interaction between NaF and ceramide (*p* = 0.846). Data represent the mean ± SEM from five independent experiments using distinct membrane preparations in duplicates.

To access CerK activity, lipid phosphorylation assays were performed as described in Section 4, through the detection of C1P, as shown in Figure 2. Two spots of ceramide-1-phosphate are always observed via thin layer chromatography (TLC), formed via the phosphorylation of naturally occurring BLM ceramides, with the upper spot (Rf = 0:55 ± 0:05) and the lower spot (Rf = 0.47 ± 0.05) being referred to as ceramide species containing longer and shorter fatty acid chains, respectively. As an example, C16-ceramide comigrates with the lower spot. The experiments were performed in the absence and presence of increasing concentrations of Cer. The result presented in Figure 2A shows the same level of C1P formation (~60 fmol × mg^−1^ × min^−1^) either without or in the presence of the different concentrations of exogenous Cer used, which allows us to suggest that only endogenous Cer is available as a substrate for BLM-associated CerK. In the presence of NaF (phosphatase inhibitor), there was an increase of approximately 90% in the detection of C1P formation (~110 fmol × mg^−1^ × min^−1^), indicating the action of a C1P phosphatase associated with the BLM. This prompted us to use NaF for all lipid phosphorylation experiments aimed at C1P detection, as presented here.

**Figure 3:**
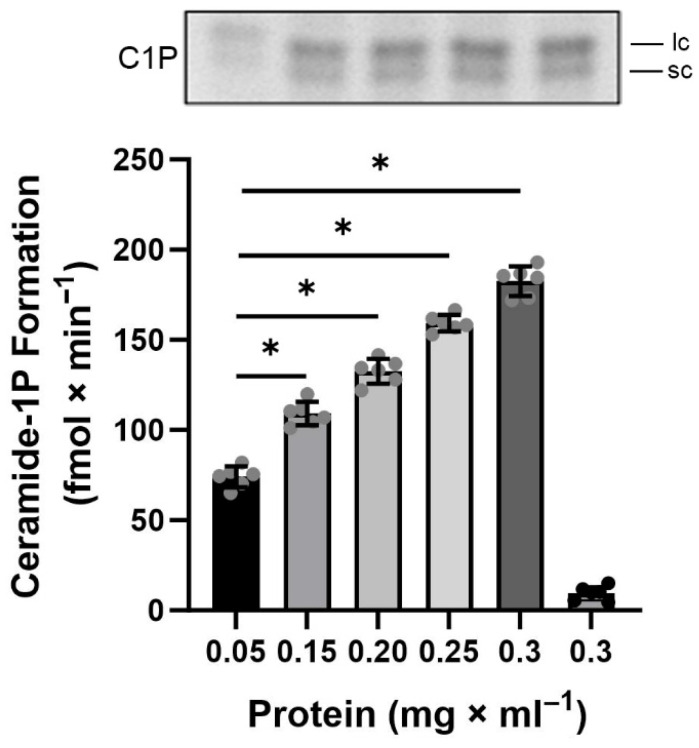
Protein dependence of CerK activity. Upper panel is a representative TLC phosphor screen autoradiogram showing the increase in ceramide-1P formation in the presence of increasing protein concentrations. The labels “lc” and “sc” on the autoradiogram refer to longer-chain and shorter-chain ceramide-1-phosphate species, respectively. Graph below shows ceramide kinase activity when basolateral membranes were incubated with increasing protein concentrations, as indicated in the abscissa. The assay was performed in the absence of exogenous ceramide and in the presence of NaF (phosphatase inhibitor). Ceramide kinase activity was measured at 20 min, 37 °C, and pH 7.2. The last bar refers to the boiled BLM fraction, resulting in the almost complete loss of CerK activity. The results are expressed as the mean ± SEM of 6 different experiments (*n* = 6) performed in duplicate, with different basolateral membrane preparations. *, *p* < 0.05 relative to the control assessed using Dunnett’s post hoc test following one-way ANOVA.

**Figure 4:**
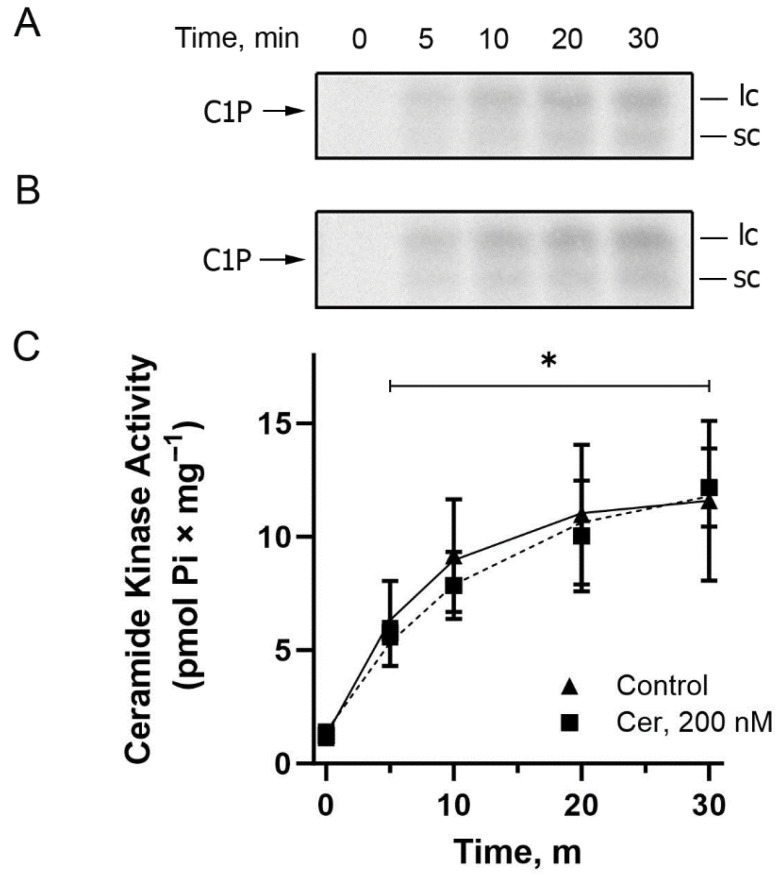
Time course of ceramide kinase (CerK) activity in basolateral membrane-enriched fractions from porcine kidney cortex. Panels (**A**,**B**) show representative TLC phosphor screen autoradiograms illustrating ceramide-1-phosphate (Cer-1P) formation over time. Panel (**A**) shows Cer-1P formation in the absence of exogenous ceramide, while Panel (**B**) shows formation in the presence of 200 nM exogenous ceramide. The labels “lc” and “sc” on the autoradiograms refer to longer-chain and shorter-chain ceramide-1-phosphate species, respectively. Panel (**C**) depicts the quantification of CerK activity over time, determined as described in Section 4, in the presence of 10 mM NaF and in the presence or absence of exogenous ceramide. Reactions were performed at pH 7.2 and 37 °C, using 0.2 mg × mL^−1^ of BLM protein. Data are expressed as the mean ± standard error from four independent experiments using different membrane preparations. Statistical analysis was performed using two-way repeated measures ANOVA, which revealed a significant effect of time (*p* = 0.0004) but no significant effect of treatment with 200 nM ceramide (*p* = 0.7094) and no significant interaction between time and treatment (*p* = 0.5918). *, time points with significantly higher Cer-1P formation relative to time zero (*p* < 0.01), based on the main effect of time.

Having demonstrated the presence of CerK activity in the BLM-enriched fractions, we proceeded to investigate the biochemical requirements necessary for maximum activity of this enzyme, beginning, for the very first time, its characterization in a well-defined compartment of renal proximal tubule cells. Initially, a curve of increasing protein concentrations ranging from 0.05 to 0.3 mg × mL^−1^ was performed, in an assay carried out for 20 min at 37 °C and pH 7.2, the maximum reaction time, temperature, and pH used in the experiments to measure ATPase activity and protein kinase activation in our previous works [3,4,5]. Figure 3 shows that the phosphorylation of endogenous Cer, by BLM-associated CerK, remained linear compared to the increase in protein concentration, and a concentration of 0.2 mg × mL^−1^ of protein was chosen for the further BLM- associated CerK characterization assays, since this was the optimal concentration used in the description of the method used for lipid phosphorylation [40], in addition to this concentration had been the protein concentration used in previous determinations of ATPase activities. Figure 4 shows a time course performed in the presence (circles) and absence (triangles) of exogenous Cer. Again, in the chosen reaction time intervals from 5 to 30 min, the same level of C1P formation was observed in the absence or presence of exogenous Cer, and 20 min was chosen for the subsequent CerK characterization experiments since this was the maximum reaction time used in the previous experiments measuring ATPase and protein kinase activities.

**Figure 5:**
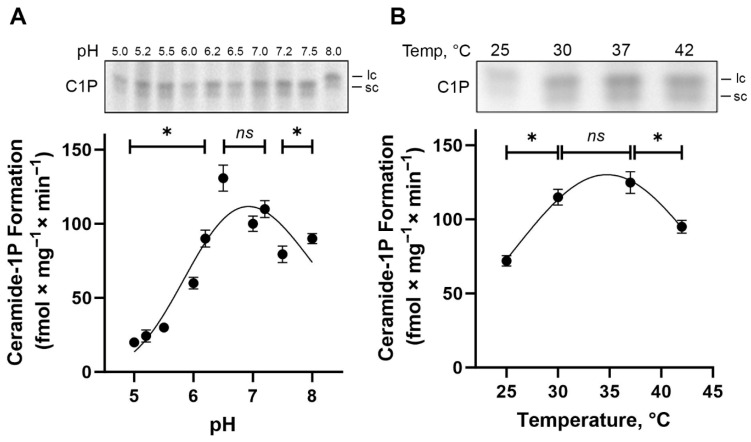
pH and temperature dependence of ceramide kinase (CerK) activity in basolateral membrane-enriched fractions from porcine kidney cortex. Panel (**A**) (top) shows a representative TLC phosphor screen autoradiogram illustrating ceramide-1-phosphate (C1P) formation at different pH values (5.0 to 8.0). The corresponding graph (bottom) shows ceramide kinase activity determined as described in Section 4, in the absence of exogenous ceramide and in the presence of 10 mM NaF (phosphatase inhibitor), at 37 °C, with 0.2 mg × mL^−1^ protein. Activity was measured at 20 min. The results are expressed as the mean ± SEM from five independent experiments using distinct membrane preparations. Data were analyzed by means of one-way ANOVA, which revealed a significant effect of pH on CerK activity (*p* < 0.0001). ns: no significant differences between pH 6.5, 7.0, and 7.2; and *: statistically significant relative to these values (one-way ANOVA with Tukey’s post hoc test). Panel (**B**) (top) shows a representative TLC phosphor screen autoradiogram of Cer-1P formation at different temperatures (25 °C to 42 °C). The corresponding graph (bottom) depicts the temperature dependence of CerK activity under the same assay conditions (absence of exogenous ceramide, presence of 10 mM NaF, pH 7.2, 0.2 mg × mL^−1^ protein, 20 min incubation). Results represent mean ± SEM from six independent experiments. A Gaussian nonlinear regression was applied to describe the temperature profile. *: significant reduction in CerK activity at 25 °C and 42 °C compared to the peak activity observed at 37 °C (*p* < 0.01); ns: no significant difference was detected between 30 °C and 37 °C (one-way ANOVA with Tukey’s post hoc test). The labels “lc” and “sc” on the autoradiograms refer to longer-chain and shorter-chain ceramide-1-phosphate species, respectively.

BLM-associated CerK activity exhibits an optimum pH between 6.5 and 7.2, leading to maximum enzyme activity and C1P formation (~120 fmol × mg^−1^ × min^−1^) (Figure 5A), revealing a highly significant effect of pH on CerK activity. The activity levels measured at pH 6.5, 7.0, and 7.2 were statistically indistinguishable (ns), indicating a plateau of maximal activity in this range, whereas all other pH values displayed significantly lower activity compared to this optimal window (one-way ANOVA with Tukey’s post hoc analysis, *p* < 0.01). Additionally, as shown in the TLC autoradiogram (Figure 5A, top panel), the formation of C1P at acidic pH (5.0–5.5), which corresponds to the optimum for acidic SMase activity, was minimal, indicating negligible contribution of SMase-driven Cer generation to C1P formation under these conditions.

To meet the requirements for BLM-associated CerK activity, the study of temperature dependence shows that CerK activity was significantly lower at 25 °C and 42 °C compared to the maximum observed at 37 °C (*p* < 0.01), while no significant difference was found between 30 °C and 37 °C (ns) (Figure 5B). This observation matches the Gaussian distribution applied to model the temperature response curve. This fitting identified a peak activity centered at 34.73 °C, highlighting that CerK activity is optimized within a narrow physiological temperature window. As expected, boiled BLM fractions completely lost the capacity to form C1P.

**Figure 6:**
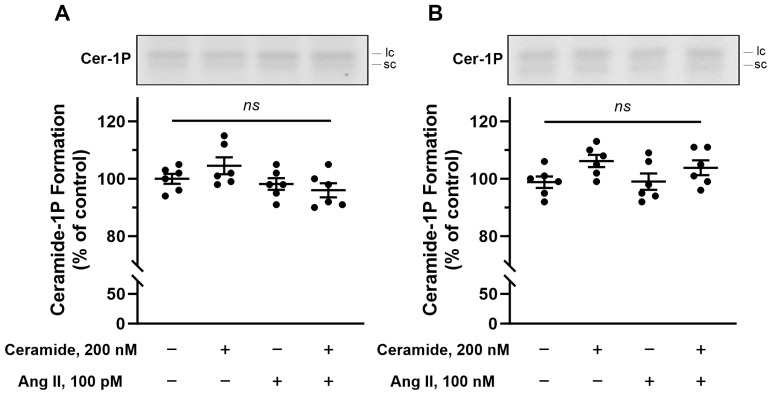
Angiotensin II does not increase ceramide-1-phosphate (ceramide-1P) formation in renal basolateral membranes. Representative TLC phosphor screen autoradiograms showing ceramide-1P formation are displayed above each corresponding graph. The labels “lc” and “sc” on the autoradiograms refer to longer-chain and shorter-chain ceramide-1-phosphate species, respectively. Panel (**A**) shows the effect of Angiotensin II (Ang II) at 100 pM, and Panel (**B**) shows the effect at 100 nM. In both experiments, ceramide kinase activity was determined as described in Section 4, using basolateral membrane preparations incubated with ceramide (200 nM) in the presence or absence of Ang II, as indicated on the abscissas. All assays were performed in the presence of NaF. The labels sc and lc on the autoradiograms refer to short-chain and long-chain ceramide-1-phosphate species, respectively. Results are expressed as the mean ± standard error (*n* = 6). “ns”, no statistically significant differences between conditions as tested by means of one-way ANOVA (Panel (**A**), *p* = 0.096; Panel (**B**), *p* = 0.108).

**Figure 7:**
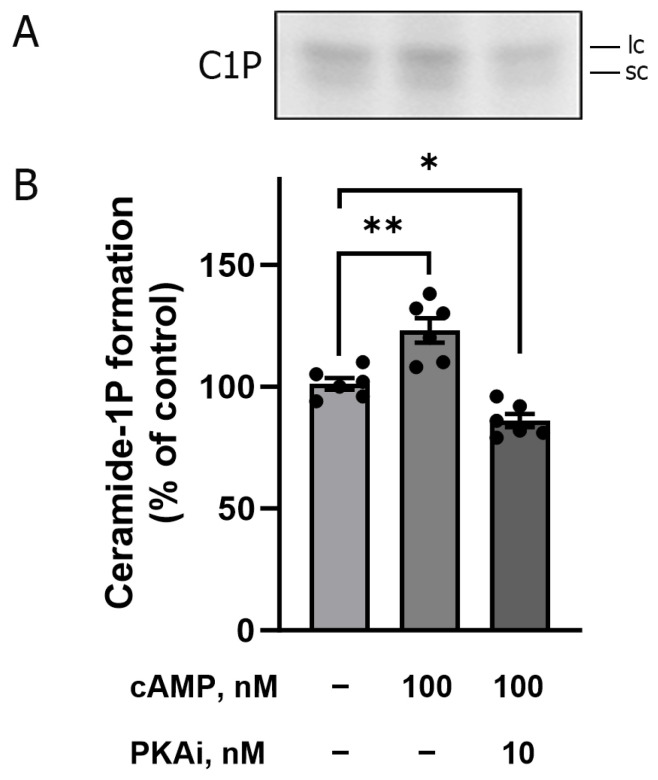
Increased formation of ceramide-1P in the presence of cAMP. Panel (**A**) is a representative TLC phosphor screen autoradiogram showing increased formation of ceramide-1P. The labels “lc” and “sc” on the autoradiogram refer to longer-chain and shorter-chain ceramide-1-phosphate species, respectively. Ceramide kinase activity was measured as described in Section 4. The experiments were performed in the presence and absence of cAMP and PKAi, in the combinations indicated on the abscissa of the figure (Panel (**B**)). The assay was performed in the presence of NaF (phosphatase inhibitor). The results are expressed as the mean ± standard error of 6 different experiments performed, with different basolateral membrane preparations. *, *p* < 0.05 and **, *p* < 0.001 One-Way ANOVA followed by Tukey’s post hoc test indicating the different levels of C1P formation compared to the control.

The next set of experiments aimed to show that the BLM-associated CerK would be targeted by bioactive compounds known to modulate different renal processes that depend on BLM harboring molecular machinery. Initially, we incubated the BLM with circulating concentrations of angiotensin II (100 pM), since this autacoid has, in addition to its known renal action, an action in increasing Cer levels in rat pheochromocytoma cell lines [41]. However, no increase in C1P formation was observed when the BLM were incubated with 100 pM or 100 nM angiotensin II alone (see the third bar in Figure 6A,B) or in the presence of 200 nM exogenous Cer (Cer concentration that activates Ca^2+^-ATPase [3] and inhibits Na^+^-ATPase [4] (see the fourth bar in Figure 6A,B). Next, we look for an already known activator of SMases, as our results suggest that only the BLM endogenous Cer would be available to CerK. Parathyroid hormone (PTH) matches this point as it was shown to increase Cer levels and stimulate Ca^2+^ influx via the cAMP/PKA axis in endothelial cells [42]. Based on these data, we decided to evaluate the involvement of the cAMP/PKA pathway in the increase in BLM C1P formation. The increase in cAMP levels (classical PKA activator) leads to the activation of PKA, with probable activation of acidic and neutral SMases, which would increase the levels of endogenous Cer, leading to the activation of CerK and an increase in C1P formation within BLM. To test this hypothesis, we incubated the BLM with 100 nM cAMP and then performed a phosphorylation and lipid extraction assay. As previously suggested, an increase in C1P formation was observed in the presence of cAMP (see second bar, Figure 7B. This activation was reversed when the BLM fractions were pretreated with 10 nM PKAi (PKA inhibitor) before the addition of cAMP (see third bar of Figure 7B). These results, shown in Figure 7, clearly demonstrate the involvement of PKA (triggered by higher levels of cAMP) in the C1P generation pathway present in the BLM.

**Figure 8:**
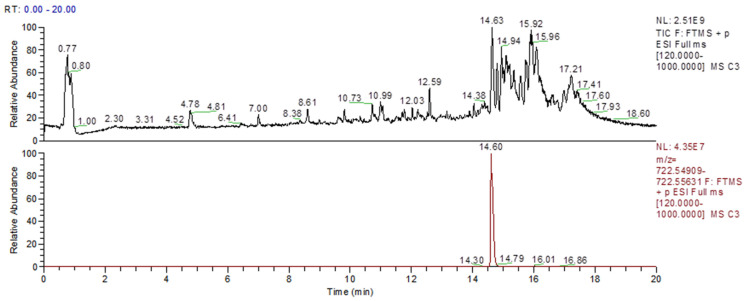
Untargeted lipidomics showed basal levels of C1P in HK-2 cells. Cells were cultured and prepared as described in Section 4. Representative sample from which C1P was extracted and identified. The upper panel shows the full-scan analysis, while the lower panel highlights an example of C1P (18:1/24:4) *m*/*z* [M+H]+ 722.5527 identified at a retention time of 14.6 min. Experiment performed in triplicate, *n* = 3.

Liquid chromatography-high resolution mass spectrometry analysis (LC-HRMS) was used to investigate the ability of renal proximal tubule living cells (HK-2 lineage) to produce C1P using a routine experimental protocol [43,44]. This allowed us to demonstrate physiologically the Cer/CerK/C1P pathway. Figure 8 shows an untargeted lipidomic analysis for whole HK-2 cell homogenates, showing that in basal conditions, a peak at the same retention time as the C1P with a mass error below 5 ppm indicates the presence of C1P in the homogenate (Figure 8). As a limitation of the study, we were not able to precisely quantify the C1P levels, but further experiments focused on the different Cer species present and the way they are remodeled within human kidney proximal cells, even in different renal injury protocols in vitro, are on the way to clarify this point.

**Figure 9:**
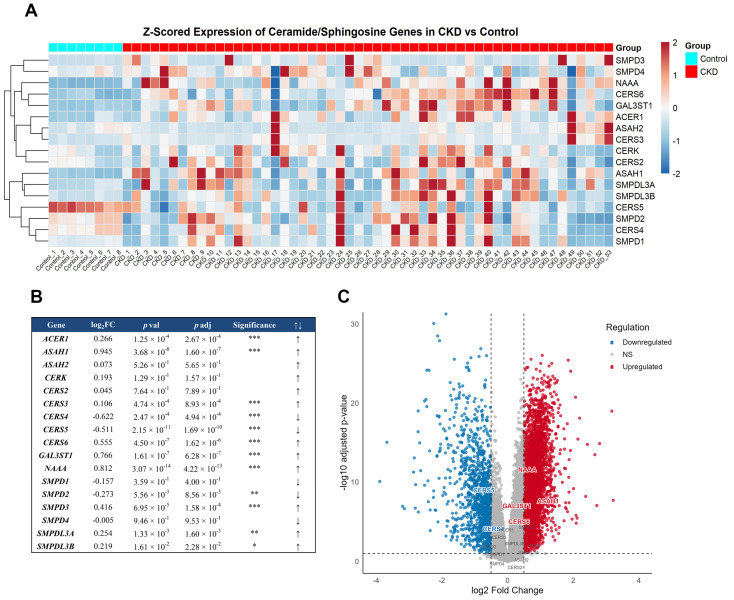
Differential expression of sphingolipid metabolism genes in CKD. (Panel (**A**)) Heatmap of z-scored expression values for ceramide and sphingosine metabolism-related genes in control (*n* = 8) and CKD (*n* = 53) kidney samples. (**B**) Statistical summary table showing log_2_ fold change (log_2_FC), *p*-values, FDR-adjusted *p*-values (*p*adj), significance levels (* *p* < 0.05; ** *p* < 0.01; *** *p* < 0.001), and expression direction (↑ upregulated, ↓ downregulated) for key genes based on Welch’s t-test with Benjamini–Hochberg correction. (**C**) Volcano plot based on the same Welch *t*-test. Genes are colored according to significance: red for upregulated, blue for downregulated, and grey for non-significant changes. Vertical dashed lines indicate log_2_FC thresholds (±0.5), and the horizontal dashed line indicates the FDR threshold (*p*adj = 0.05).

To complement our biochemical and cellular evidence of a functional sphingolipid signaling pathway in kidney proximal tubule basolateral membranes, we investigated whether the transcriptional regulation of key enzymes involved in Cer and C1P metabolism is altered in chronic kidney disease (CKD). A transcriptomic analysis of the GEO GSE66494 dataset [45,46] revealed a distinct transcriptional remodeling of genes involved in sphingolipid metabolism in CKD kidneys compared to controls (Figure 9). The heatmap analysis revealed a distinct transcriptional remodeling of genes involved in sphingolipid metabolism in CKD kidneys compared to control samples (Figure 9A). Notably, N-acylsphingosine amidohydrolase 1 (ASAH1; log_2_FC = 0.945, *p*adj = 1.6 × 10^−7^), N-acylethanolamine acid amidase (NAAA; log_2_FC = 0.812, *p*adj = 4.2 × 10^−13^), and galactose-3-O-sulfotransferase 1 (GAL3ST1; log_2_FC = 0.766, *p*adj = 6.3 × 10^−7^), enzymes associated with Cer degradation and sulfatide metabolism, were significantly up-regulated. In parallel, ceramide synthase 4 (CERS4; log_2_FC = −0.622, *p*adj = 4.9 × 10^−4^) and ceramide synthase 5 (CERS5; log_2_FC = −0.511, *p*adj = 1.6 × 10^−10^), which are responsible for Cer biosynthesis, showed significant downregulation, whereas ceramide synthase 6 (CERS6; log_2_FC = 0.555, *p*adj = 1.6 × 10^−6^) was upregulated (Figure 9B,C). This pattern suggests a shift toward Cer turnover favoring sphingosine and sulfatide pathways. This is further supported by the volcano plot (Figure 9C), which clearly highlights the most significantly altered genes in CKD, with multiple sphingolipid-related genes surpassing both the log_2_ fold-change and FDR thresholds. These findings reinforce the notion that the Cer/CerK//C1P metabolic axis undergoes adaptive regulation during kidney injury.

## 3. Discussion

The different transport processes present throughout the nephron make each segment a specialized compartment. Among the nephron segments, the proximal tubule accounts for almost 60–65% of the reabsorption of glomerular fluid substances [9,10,47]. Nephron epithelium cells have some specific characteristics when compared to those of other tissues, highlighting their morphological and functional polarity [44,47,48]. The plasma membrane of these cells has two very distinct regions: an apical portion (focused on the tubular lumen) and another basolateral portion facing the renal interstitium with differential distribution of transporters, surface proteins, receptors, lipids, and effector proteins (e.g., protein kinases and phospholipases). Various groups, including ours, contribute to continually updating the adaptive repertoire of cellular signaling elements and processes that form the foundation for normal kidney function. In the present paper, we included a new part of this complex puzzle: a BLM-associated machinery ready for quick response that led to sphingolipid turnover resulting in C1P formation within the BLM moiety. It has been described in the literature that some lipids play a key role in regulating membrane dynamics. For example, it has been demonstrated that very low proportions of ceramides, diacylglycerol, some fatty acids, sphingolipids, glycolipids, and their metabolic products cause changes in the molecular organization of mixed interfaces with phospholipids. This can extend to supramolecular levels and induce topological changes that affect the formation of segregated domains of different phase states, thickness, and interfacial curvature that can participate in signal transduction mediated by phospholipase A2 (PLA2), phospholipase C (PLC), and SMases [11,12,13,14]. In the present paper, we evidenced the machinery harbored at the BLM, which includes the SMases and the quick and possible coupled action of CerK, providing local augmentation in C1P levels, thus acting in a modulatory balance between Cer and C1P that results in physical chemical membrane structure alterations and cellular signaling, respectively.

Sphingomyelin is one of the most abundant lipids present in the apical membrane of renal proximal tubule cells and is also present in the BLM, with it being found preferentially on the external face of the lipid bilayer, forming hydrophobic interactions with cholesterol and giving these membranes less fluidity [32]. It was classically demonstrated that changes in the fluidity and/or lipid composition of the plasma membrane affect the activity of co-transporters present in the apical membrane. SM degradation inhibits the Na^+^/glucose and Na^+^/Pi co-transporters present in the apical membrane of renal epithelial cells, regardless of changes in membrane fluidity [49]. Our group has previously investigated the involvement of Cer and its phosphorylated derivative, C1P, using the same biological model presented here [3,4,5].

The study of the presence of SMases in the BLM becomes important because: i. They generate Cer, which leads to significant changes in the organization of the plasma membrane, favoring the formation of Cer-rich nano and microdomains, which are capable of spontaneous fusion with other microdomains, thus resulting in the so-called signaling platforms triggering several cellular responses [36,37,38]; ii. They are responsible for the accumulation of Cer in the proximal tubules during ischemia or other types of renal injury, with SMases being important pharmacological targets [49,50]. It is important to remember that the increase in endogenous Cer levels occurs through the activation of acidic and/or neutral SMases present in the BLM (Figure 1).

Structural studies show that membrane-associated neutral sphingomyelinases form stable dimers via interactions mediated by their C-terminal transmembrane domains [51]. Our Western blot analysis (Figure 1) revealed a strong band at ~145 kDa detected by the anti-nSMase antibody, consistent with the dimeric form of SMPD3 (nSMase2). This aligns with structural studies showing that SMPD3 forms stable homodimers via its C-terminal transmembrane domains, which are crucial for membrane association and enzymatic function [51,52]. The localization of SMPD3 in this fraction is further expected based on its known targeting to the plasma membrane and enrichment in detergent-resistant microdomains [53]. The localization of SMPD3 in caveolae-enriched BLM, as previously demonstrated for this BLM preparation [54], likely stabilizes this dimeric form within detergent-resistant lipid microdomains involved in signaling [53]. The observed band pattern indicates that the nSMase is not only highly expressed in BLM but also likely maintained in an oligomeric (dimeric) state within these detergent-resistant microdomains. In contrast, the weak ~70 kDa signal detected by the anti-aSMase antibody corresponds to SMPD1, a lysosomal enzyme present only as a minor contaminant. This further confirms the plasma membrane enrichment of the BLM fraction and supports that the SMase activity relevant to our model predominantly arises from SMPD3.

Studies in the literature show that Cer is considered a direct activator of atypical PKC ξ (zeta); this activation is involved in cell proliferation and activation of MAPK cascades [55,56]. In renal mesangial cells, it was observed that Cer can also activate other PKC isoforms, known as PKCα and βII (classical) and PKCε, θ, and η (novel) [57], which also gives Cer an important role in the regulation of PKC-mediated events. Protein kinases, known as PKA and PKC, have been associated with the modulation of PMCA [3,6], renal proximal tubule Na^+^-ATPase [7,58,59], and other ion transporters [60,61] from different stimuli in different biological models, including renal tissue. Furthermore, data from the literature have shown that ceramides can activate protein kinases, leading to responses such as proliferation, apoptosis, phagocytosis, inflammation, etc. [16,17,24,62].

Upon the rise in membrane Cer content resulting in different cellular responses, it would be necessary to “clean” the excess of the sphingolipid either through the action of CDases or CerK, with the second being highly relevant, as it brings to the scene another bioactive sphingolipid, C1P, which was only considered of physiological relevance years after the CerK identification. CerK was only cloned in 2002 [25], which can explain the limited literature about the activity of this enzyme. As a limitation of this work, we could not demonstrate through Western blotting the detection of CerK within the BLM or HK-2 cells, with the commercially available antibody used being more appropriate for immunohistochemistry, which was not explored here. The results presented herein shed light, at least in part, on some of the questions about CerK activity and modulation.

The ability of Cer and C1P to modulate Na^+^ and Ca^+^ active transporters through selectively triggering different protein kinases associated with the BLM allows us to classify this bioactive lipid axis (SM/SMases/Cer/CerK/C1P) as an important player in renal physiology. This would also be true if we consider pathophysiological alterations as those classically highlighted by different authors to consider the Cer generation pathway from SM responsible for early events in kidney diseases [50,63,64,65]. Thus, the kinase-mediated modulation of the BLM active transporters by Cer and C1P could be especially relevant during renal injury. It has been demonstrated that Cer levels in the kidney are elevated owing to an increased Ca^2+^-stimulated sphingomyelinase activity after cellular Ca^2+^ homeostasis is disrupted. Non-targeted lipidomic analysis for C1P in HK-2 cells showed that this bioactive lipid is present in control conditions [43,44]. Further experiments are on the way to precisely identify and quantify all the molecular species of Cer and C1P, which would allow us to precisely detect altered patterns of these ceramides when submitting HK-2 cells to different in vitro injury protocols, as mentioned above.

Our transcriptomic dataset analysis strongly supports this view, revealing a coordinated transcriptional remodeling of the sphingolipid metabolic network in CKD. The upregulation of CDases ASAH1 and NAAA and the downregulation of key ceramide synthases CERS4 and CERS5 suggest an adaptive cellular strategy to modulate Cer levels and maintain membrane homeostasis under injury conditions. ASAH1 is a major regulator of Cer–Sph balance, and its dysregulation has been shown to drive Cer accumulation, promoting apoptosis and fibrosis in renal disease contexts [66,67,68]. Additionally, CERS5–mediated C16-ceramide generation has been implicated in hypoxia-induced apoptotic signaling, a hallmark of tubular injury [69,70]. This shift toward enhanced Cer turnover could potentially favor the generation of Sph and its phosphorylated derivatives while modulating C1P dynamics. This mirrors the biochemical observations of CerK activation in BLM fractions and supports the notion that the Cer/CerK/C1P axis is dynamically regulated during renal stress.

It is important to remember that the increase in endogenous Cer levels occurs through the activation of acidic and/or neutral SMases present in the BLM (Figure 1), thus providing the substrate for CerK activity, resulting in C1P. Despite the presence of both acidic and neutral SMases in the BLM, acidic SMases require an acidic pH (between 4.5 and 5.5) to be fully activated. For this reason, aSMases are probably sub-activated in the experimental conditions for the phosphorylation and lipid extraction assays, which were performed at neutral pH (pH 7.2). In other words, as a limitation of our work, the action of acidic SMases was possibly underestimated under these conditions. Neutral SMases would also be under-activated, since the enzyme is partially inhibited by Ca^2+^ and EDTA [71], both present in the reaction medium used to measure CerK activity, since CerK is described as a Ca^2+^-dependent enzyme [40]. The fmolar concentrations of C1P detected in the phosphorylation and lipid extraction assays are probably due to the fact that the acidic and neutral SMases were not fully activated under the conditions of our phosphorylation assay, as these conditions are favorable only for the best functioning of CerK (phosphorylating Cer into C1P), which was our main interest. We can postulate that the joint action of the acidic and neutral SMases present in the BLM leads to an increase in the formation of endogenous Cer and consequently to a greater increase in C1P levels, reaching concentrations that could result in the modulation of different ion transporters harbored in the BLM.

To find a physiological activator of the BLM-associated SMases and CerK signaling pathways presented here, we searched the literature for a hormone that modulates the signaling pathway involving ceramides. We found that: i. parathyroid hormone (PTH) leads to the activation of adenylate cyclase and formation of cAMP [72]; ii. PTH leads to an increase in Cer concentrations and stimulates Ca^2+^ influx in endothelial cells [42]; iii. Cer stimulates Ca^2+^ reabsorption in the BLM through the activation of PKA and consequent activation of PMCA [3]. Based on these data, we decided to evaluate the involvement of the cAMP/PKA pathway in the modulation of CerK activity. The result presented in Figure 7 clearly shows the involvement of cAMP/PKA in the stimulation of C1P production within the BLM, as it was completely abolished when the BLM fractions were pre-incubated with 10 nm PKAi (PKA inhibitor) before the addition of cAMP (see third bar in Figure 7B). Based on these findings, we can hypothesize that different hormones and autocoids with PKA-dependent renal actions could be potential candidates to play key roles in the ceramides generation pathway in the BLM. Thus, we would have two possibilities: (1) PKA would activate the SMases, increasing endogenous Cer levels, which would enable higher amounts of C1P formation by Cerk, or (2) PKA phosphorylation of CerK, promoting an increased activity of the enzyme with further increased C1P formation. Our results do not enable us to definitively identify one of these alternatives; however, the first option appears plausible. This pathway, which involves stimulatory G-protein-coupled receptors, has already been demonstrated to preferentially activate SMases. This activation leads to the generation of Cer within the plasma membrane, contributing to the formation of ceramide-rich membrane microdomains [36] or the production of C1P through CerK activation, as demonstrated in our findings.

Among these possible modulators, we can suggest not only PTH, already known to increase Cer levels in other cell types, as well as angiotensin (1–7), which is known to modulate important ion fluxes through the BLM in a PKA-dependent manner [1]. Curiously, AngII, which is an autacoid with important PKC-dependent actions in renal proximal tubule cells, was not able to modulate C1P generation in the experimental conditions described for Figure 6, either in the absence or presence of exogenous Cer.

The hydrocarbon chain of ceramides can vary in size, leading to different cellular responses. The presence of both short-chain and long-chain C1P was clearly observed under the applied conditions (Figure 3). Our group is advancing in lipidomics analysis to characterize the Cer species generated and correlate them to health or disease, aiming to explore their potential as a versatile biomarker for different diseases [17], including nephropathies.

## 4. Materials and Methods

### 4.1. Material

Buffers, BSA, and protease inhibitors were obtained from Sigma Chemical Co. (St. Louis, MO, USA), and Percoll was from Pharmacia (Peapack, NJ, USA). Distilled water, deionized using the Milli-Q system of resins (Millipore Corp., Burlington, MA, USA), was used to prepare all solutions. ^32^P_i_ was obtained from the Instituto de Pesquisas Energéticas e Nucleares (IPEN, São Paulo, Brazil). [γ-^32^P]ATP was prepared as described previously [3]. Cer (from bovine brain), C1P, PKA α-catalytic subunit inhibitor 5–22 peptide (PKAi) C were purchased from Sigma Chemical Co. All other reagents were of the highest purity available. Nitrocellulose membranes (Hybond, Escondido, CA, USA) and the ECL^TM^ system were from GE Healthcare (Chicago, IL, USA).

### 4.2. Isolation of Basolateral Membranes (BLM)

Pig kidneys were purchased from a licensed slaughterhouse (Frigorífico Novo Meriti, São João de Meriti, RJ, Brazil) at the moment of the pig slaughtering. The chosen slaughterhouse is in accordance with the Brazilian Federal Law No. 5.760, which establishes the requirements for this activity, including the supervision of the processes by a veterinarian. Kidneys were transported in a chilled solution containing 250 mM sucrose, 10 mM Hepes–Tris (pH 7.6), 2 mM EDTA, 1 mM PMSF, and 0.15 mg/mL of soybean trypsin inhibitor. The external portion of the cortex (*cortex corticis*) was carefully removed, and enriched basolateral membranes (BLM) fractions derived from kidney proximal tubules were prepared using the Percoll gradient method [73]. Controls for contamination with other membranes were carried out as previously described [3,5]. The specific activity of the basolateral membrane marker Na^+^ + K^+^−ATPase (297.2 ± 1.2 nmol/mg per min) was enriched six-fold over the initial kidney cortex homogenate. The membranes were stored in 250 mM sucrose in liquid N_2_.

### 4.3. Cell Cultures and Treatments

Human renal proximal tubule cells (HK-2, ATCC: CRL:2190) were cultured in KSF-M medium (Keratinocyte Serum Free Medium, Thermo Fisher Scientific, Waltham, MA, USA) supplemented with 1% pen/strep (Thermo Fisher Scientific) and 2% Fetal Bovine Serum (FBS, Cultilab, São Paulo, Brazil). Every 4 days, cells were trypsinized (0.25%, Thermo Fisher Scientific) using the following proportion: 1:4, and cells were maintained under 5% CO_2_ at 37 °C to achieve 80–90% confluency prior to the experiments [43,44].

### 4.4. Protein Determination

The protein concentration of basolateral membrane preparations was classically determined by the Folin phenol method [74], using bovine serum albumin as a standard.

### 4.5. Western Blotting

Protein lysates (100–200 µg) from basolateral enriched membrane fractions were separated by 10% SDS-PAGE and transferred to nitrocellulose membranes (0.2 µm; Bio-Rad, Hercules, CA, USA). Membranes were blocked for 1 h in TBS-T (20 mM Tris, 137 mM NaCl, 0.1% Tween-20, pH 7.6) containing 5% non-fat dry milk, followed by overnight incubation at 4 °C with primary antibodies diluted in TBS-T/1% milk: goat anti-acid sphingomyelinase (SMPD1; Novus Biologicals, Centennial, CO, USA, IC4048G; 1:1000) or rabbit anti-neutral sphingomyelinase (SMPD3; Novus Biologicals, NBP3-23413; 1:1000). After washing, membranes were incubated for 1 h with HRP-conjugated donkey anti-goat IgG (1:10,000) or donkey anti-rabbit IgG (1:10,000) (A15999; Thermo-Fischer Scientific), washed again, and detected with Pierce™ ECL substrate using a ChemiDoc™ XRS+ imaging system (Bio-Rad).

### 4.6. Phosphorylation Assay and Lipid Extraction from the BLM Fraction

Total lipids were extracted from the BLM fractions as previously described [3,40], which starts with the isolated membrane (0.2 mg of final protein) incubated for 20 min at 37 °C in a reaction medium (0.5 mL) containing: [ƴ-^32^P]ATP 10^8^ cpm, MOPS-Tris 20 mM (pH 7.2), NaCl 50 mM, DTT 1 mM, EDTA 2 mM, CaCl_2_ 3 mM, NaF 10 mM, MgCl_2_ 100 mM, ATP-Na^+^ 1 mM. Exogenous Cer was added when referred to in the figures, as indicated.

The reaction was stopped with 8.6 mL of a chloroform–methanol solution (1:1 *v*/*v*), and then 3.6 mL of MOPS-KCl (1 M) was added. The phases were separated by centrifugation at 1000× *g* for 10 min at room temperature. The organic (lower) phase was transferred to a new tube, and the solvent was evaporated under nitrogen. The free Ca^2+^ concentration for these determinations was calculated using a computer program that took into account the different species involved in the equilibrium between EGTA, Ca^2+^, the different ATP forms, Mg^2+^, H^+^, and K^+^, as already described [75]. Each condition was tested in independent replicates as indicated in the figure legends, and the resulting data were subsequently analyzed as described in Section 4.11.

### 4.7. Lipid Separation and Identification

The extracted lipids were separated and analyzed by thin-layer chromatography (TLC) with one-dimensional running on silica gel chromatoplates (60 F254, Merck, Rahway, NJ, USA), using a solvent system containing chloroform–acetone–methanol–acetic acid–water (10:4:3:2:1 *v*/*v*) [40]. The chromatoplates were previously activated at 110 °C for 10 min, and then the entire volume of the lipid extract was applied, together with standard lipids. After the running and complete evaporation of the solvents, an X-ray film (Kodak X-Omat™) or the “phosphor screen” (PhosphorImager Storm 860—Molecular Dynamics, Amersham Biosciences, Sunnyvale, CA, USA) was exposed to the chromatoplate. After 48 h, the film or phosphor screen was developed, and the chromatoplate was exposed to iodine vapors to visualize the lipids, enabling the determination of the relative mobility of each lipid in the different samples and the C1P standard (10 µg) applied. The areas corresponding to C1P were marked on the chromatoplate, overlapping them with those of their respective autoradiogram.

To quantify the radioactive presence in the marked C1P, the silica from the region of the chromatoplate containing the radioactive lipid spot was carefully scraped with a scalpel and placed directly into vials containing a scintillator solution composed of toluene containing 200 mg/L of POPOP (1,4-bis [5-phenyl-2-oxazolyl]-benzene; 2,2′-p-phenylene-bis [5-phenyloxazole]). Radioactivity was measured in a Packard Tri-Carb 2100 TR liquid scintillation counter (Palo Alto, CA, USA) and calculated from the nanomoles of Pi used in the reaction. The quantified C1P levels were used to calculate enzyme activity in pmol Pi × mg^−1^ × min^−1^, and were subjected to statistical analysis as described.

### 4.8. Sample Preparation for LC–MS/MS Analysis

After the experimental set, HK-2 cells were washed twice with phosphate-buffered saline (PBS) and then dissociated from the culture flasks with trypsin (0.25%). The suspended cells were centrifuged at 1000 rpm for 5 min to remove trypsin, and the cells were resuspended in 400 μL of methanol and 100 μL of water. The mixture was vortex-mixed for 10 s, followed by centrifugation at 12,000 rpm for 5 min, and then the supernatant was transferred to clean vials. The extracts were taken to dryness under a gentle stream of nitrogen and redissolved in a small volume (50 μL) of acetonitrile, isopropanol, and water (1:2:1) for analyses.

### 4.9. Liquid Chromatography-High Resolution Mass Spectrometry Analysis

The LC-HRMS system consisted of a Thermo Scientific UltiMate 3000 LC system coupled to a Thermo Scientific Q Exactive Plus Quadrupole-Orbitrap Mass Spectrometer equipped with an electrospray (ESI) ion source. Chromatographic separation was obtained using a Waters CSH C18 column (150 mm × 2.1 mm × 2.5 µm) at 45 °C. The mobile phase consisted of (A) acetonitrile–water (60:40, *v*/*v*) with 0.1% formic acid and 10 mM ammonium formate and (B) isopropanol–acetonitrile (90:10, *v*/*v*) with 0.1% formic acid and 10 mM ammonium formate. Analytes were separated using an elution gradient as follows: 0.0 min 50% B; 10.0 min 100% B; 10–15 min 100% B; 15.1 min 50% B; 20 min 50% B. The flow rate was 400 µL/min. The mass spectrometer was operated in the positive ion mode (ESI+) with a capillary voltage of 3900 V, S-lens RF level of 70 (arbitrary units, a.u.), and capillary temperature of 320 °C. The sheath and auxiliary gas flow rates (nitrogen) were 45 and 20 (a.u.), respectively. Samples were analyzed in the scan range of *m*/*z* 120–2000 at the resolution of 70,000 FWHM (full width at half maximum) followed by data-dependent MS/MS analysis (ddMS2 top3 experiment, normalized collision energy (NCE) 25%). Data acquisition was performed on Xcalibur 3.0 software (Thermo Scientific). LC-HRMS data were imported to MS-Dial 5.5 software for data processing. Positive MS spectra were used. Lipid annotation was performed by comparing the *m*/*z* of the precursor ion with an error <5.0 ppm and MS/MS spectra with those of the MS-Dial LipidBlast library.

### 4.10. Statistical Analysis of Differential Expression from Transcriptomic Datasets

Publicly available transcriptomic data were retrieved from the Gene Expression Omnibus (GEO) database under the accession number GSE66494, originally published and deposited [45,46]. This dataset comprises gene expression profiles derived from renal biopsy specimens of patients with chronic kidney disease (CKD) (N = 53) and control (N = 8) individuals. The microarray data containing probe-level intensity values quantile-normalized to the median across all arrays was analysed in the R environment (version 4.3.1) using RStudio (version 2025.05.1+513). The data was processed using the Agilent GPL6480 annotation, which maps probe identifiers to gene symbols. Probe-to-gene conversion was performed using the Entrez Gene ID and Ensembl Gene ID fields from the platform annotation, followed by collapsing multiple probes per gene by mean expression. Gene differential expression analysis was performed using the Welch *t*-test, which accounts for unequal variances between groups, and *p*-values were adjusted using the Benjamini–Hochberg (FDR) correction. Genes were classified as significantly differentially expressed if the adjusted *p*-value (*p*adj) < 0.1 and presented an absolute log2 fold change (|log_2_FC|) > 0.5. The R packages ggplot2 v 3.5.1 and ggrepel v 0.9.6 were used to generate a volcano plot highlighting the significantly upregulated (red) and downregulated (blue) genes among all annotated genes in the dataset. A heatmap was generated with the pheatmap package v 1.0.12 to show Z-score standardized expression profiles of ceramide and sphingosine metabolism-related genes across samples.

### 4.11. Statistical Analysis

Data are expressed as the mean ± standard error of the mean (SEM) from the indicated number of independent experiments. Statistical analyses were performed using GraphPad Prism version 10.1.2 (GraphPad Software, Boston, MA, USA). Differences between experimental conditions were evaluated using one-way ANOVA or two-way repeated measures ANOVA, as appropriate, followed by Tukey’s post Hoc multiple comparisons test. Nonlinear regression was applied to model pH dependence (log-normal fit) and temperature dependence (Gaussian fit) of ceramide kinase activity. The quality of curve fits was assessed by the coefficient of determination (R^2^). The temperature dependence fit was modelled using a Gaussian equation, yielding a peak (mean) at 34.73 °C (95% CI: 34.40 to 35.09), an amplitude of 130.2 (95% CI: 126.0 to 134.4), and a standard deviation (SD) of 9.043 (95% CI: 8.510 to 9.679), with a goodness-of-fit R^2^ = 0.9424. The temperature values tested were 25 °C, 30 °C, 37 °C, and 42 °C. A significance threshold of *p* < 0.05 was adopted for all analyses.

## 5. Conclusions

From the results presented herein, a signaling cascade starting with Cer production and further phosphorylation to C1P is an efficient pathway for modulating different transporters in renal proximal tubules, which are the nephron segment where more than two-thirds of the glomerular ultrafiltrate is reabsorbed. The above results add evidence to the view that Cer and C1P participate in the regulatory network of bioactive sphingolipids and glycerolipids resident in this nephron segment, which should be true for the overall nephron. The biochemical requirements associated with the BLM-associated CerK may be conserved in CerK from other tissues and subcellular compartments; thus, it will be helpful to consider this molecular machinery another promising drug target for different diseases.

## Data Availability

Data supporting the reported results can be found in the original files at the Biomembranes Laboratory.
